# Cannabidiol-induced Heme oxygenase-1 contributes to modulate the phenotype of hiPSC-derived cardiac fibroblasts from patients with Duchenne muscular dystrophy

**DOI:** 10.1016/j.redox.2026.104333

**Published:** 2026-08-05

**Authors:** L. Savchenko, S. Soussi, D. Rovina, A. Swiader, S. Mallia, G. Pompilio, O. Kunduzova, A. Parini, A. Gowran, N. Pizzinat

**Affiliations:** a Institut des Maladies Métaboliques et Cardiovasculaires I2MC, Toulouse, France; b Unit of Inherited Cardiomyopathies, Centro Cardiologico Monzino IRCCS, Milan, Italy; c Department of Biomedical, Surgical and Dental Sciences, University of Milan, Italy

**Keywords:** Duchenne muscular dystrophy, Cannabidiol, Heme oxygenase-1, Phenotypic modulation

## Abstract

Duchenne muscular dystrophy (DMD) is a severe and progressive form of muscular dystrophy caused by mutations in the dystrophin gene. We previously observed that loss of dystrophin in human induced pluripotent stem cell–derived cardiac fibroblasts (hiPSC-cFib) dysregulated the actin network and induced a metabolic remodeling associated with an exacerbated myofibroblast phenotype. The endocannabinoid signaling (ECS) system plays an important role in chronic inflammatory and fibrotic conditions and is dysregulated in skeletal muscle of DMD patients. Here, we investigated the effects of cannabidiol (CBD) on hiPSC-cFib from healthy controls and DMD patients. CBD failed to modify metabolic responses in DMD hiPSC-cFib, while significantly promoting glycolysis and cell proliferation in control hiPSC-cFib. Despite these distinct metabolic responses, CBD significantly attenuated TGF-β–induced myofibroblast activation in both DMD and control hiPSC-cFib by lowering α-smooth muscle actin and collagen type I levels suggesting a metabolism-independent mechanism. Additionally, CBD exerted strong antioxidant effects on both DMD and control hiPSC-cFib, markedly reducing intracellular reactive oxygen species (ROS) levels, increasing GSH levels and robustly inducing heme oxygenase-1 (HO-1) expression in a time- and dose-dependent manner which could not be mimicked by CB1R or CB2R agonists and blocked by their antagonists**.** Pharmacological inhibition of HO-1 blunted CBD's ability to suppress TGF-β–induced activation of DMD and control hiPSC-cFib, demonstrating that HO-1 is a key mediator of CBD's anti-fibrotic action.

Together, these findings showed stimulation of glycolytic metabolism by CBD, regulation which is lost in DMD hiPSC-cFib. We uncovered a previously unrecognized HO-1–dependent pathway by which CBD dampens profibrotic activation in human DMD and control hiPSC-cFib, highlighting its potential as a therapeutic approach to limit cardiac fibrosis in Duchenne muscular dystrophy.

## 1 Introduction

Duchenne muscular dystrophy (DMD) is caused by DMD gene mutations leading to dystrophin deficiency. The loss of dystrophin results in chronic muscle damage, defective autophagy, elevated oxidative stress-induced reactive oxygen species (ROS) and inflammation [1]. Duchenne muscular dystrophy patients develop progressive muscle weakness and cardiac dysfunction [2]. Cardiomyopathy begins in the first decade of life and evolves with progressive replacement of the myocardium by fibrous and fatty connective tissue, often resulting in dilated cardiomyopathy [[3], [4], [5]]. This excessive deposition of extracellular matrix (ECM), occurring at the expense of muscle fibers and leading to progressive cardiac dysfunction, is thought to result from cardiomyocyte death [6]. Indeed, both acute and chronic cardiomyocyte injury have been shown to trigger myocardial fibrosis, a mechanism that is likely to operate in dystrophic cardiomyopathy. However, we recently demonstrated that the lack of dystrophin in human induced pluripotent stem cell–derived cardiac fibroblasts (hiPSC-cFib) from DMD patients increased cell sensitivity to pro-fibrotic stress [7]. Therefore, these dysfunctional stromal cells may directly fuel the development of scar tissue independently of cardiomyocyte loss.

Therapeutic strategies targeting different aspects of Duchenne pathology are of particular interest, as efficient treatments to correct the absence of dystrophin are not yet available. Recently cannabidiol (CBD), one of the most studied plant-derived cannabinoids, has attracted extensive attention due to its therapeutic potential, including ameliorating chronic inflammation and fibrosis formation in several disease states [8,9]. The molecular mechanisms governing the beneficial properties of this phytocompound are complex, involving allosteric modulation of the endocannabinoid receptors, regulation of oxidative status, autophagy and cell death in different pathological situations [[10], [11], [12]]. Dysregulation of the endocannabinoid system (ECS) was observed in both murine and human muscles affected by DMD [13,14]. This pro-homeostatic lipid signaling network functions trough two types of G protein-coupled cannabinoid receptor (CB1R and CB2R) [15,16]. The endocannabinoid CB1 receptor (CB1R) shows the highest degree of expression at disease onset and may exacerbate DMD myopathies since its pharmacological inhibition prevents locomotor impairment in dystrophic mice [17]. In addition, CBD can modulate the generation of ROS in different cell types [18]. In keratinocytes, it was reported to trigger expression of heme oxygenase-1 (HO-1), an essential antioxidant enzyme that regulates oxidative stress, exerts anti-inflammatory and cytoprotective effects by restoring redox balance [19]. On the other hand, high concentrations of CBD trigger mitochondrial dysfunction and ROS overproduction, reducing cell viability of different cancer cell types [20]. Here, we showed that cell metabolism of hiPSC-cFib from DMD patients have lost capacity to be stimulated by CBD. However, CBD activated HO-1 to inhibit the conversion of hiPSC-cFib into myofibroblasts revealing an unrecognized CBD–dependent pathway to dampen profibrotic activation.

## 2 Research design and methods

### 2.1 Cell culture and proliferation

As previously described, hiPSC-cFib were generated from three DMD patients carrying distinct mutations and from three healthy donors, in accordance with the Declaration of Helsinki. The healthy group comprised three healthy male donors: CON3 (50 years old at biopsy), CON4 (39 years old), and CON5 (39 years old). The DMD group included three male patients with distinct mutations in the DMD gene: DMD1 (34 years old at biopsy), harboring a deletion of exons 49–50; DMD4 (10 years old), with a deletion of exons 45–50; and DMD5 (15 years old), carrying a splice-site mutation (c.6913-1G > A). Reprogramming and iPSC generation were conducted as previously described [21]. Directed differentiation of iPSCs into epicardial-like cells (hiPSC-EPI) involved sequential exposure to CHIR99021, Activin A, BMP4, XAV939, and retinoic acid, with medium changes on Days 0, 3, and 6, followed by replating on fibronectin-coated plates and treatment with SB431542. For fibroblast differentiation, hiPSC-cFib were generated from EPI-iPSCs cultured on vitronectin-coated plates (Thermo Fisher Scientific, A14700) in fibroblast growth factor-2 (FGF-2) –supplemented BPEL medium (PeproTech, AF-100-18B) for 10 days. Cells were subsequently cultured in Fibroblast Growth Medium 3 (FGM-3; PromoCell, C-39350) containing 10% fetal bovine serum as previously described [7]. Profibrotic stimulation was performed by incubating hiPSC-cFib with transforming growth factor beta (TGF-β; 10 ng/mL) for 24 h in medium containing 0.5% serum-free supplement (SVF). Where indicated, cells were treated for 24 h with the following compounds: CBD (0.1–2.0 μM), dichloroacetate (DCA; 50–100 μM), AM251 (2 μM), AM630 (2 μM), arachidonoylethanolamide (AEA; 0.5–2.0 μM), JWH-133 (0.5–2.0 μM), chloroquine (CQ; 10 μM), N-acetyl-l-cysteine (NAC; 20 μM), or tin protoporphyrin IX (SnPPIX; 1 -5 μM).

### 2.2 Intracellular lactate levels

Intracellular l-lactate was measured using Lactate Colorimetric Assay Kit (Sigma-Aldrich) according to the manufacturer's instructions. hiPSC-cFib were seeded at a density of 1.6 × 10^6^ cells per well and treated for 24 h in 0.5% SVF in the presence of 0.5 or 2 μM CBD. Cells were collected in phosphate-buffered saline (PBS), transferred to a 96-well plate and incubated with the reaction master mix containing enzyme mix, probe, and assay buffer. After 30 min incubation, the colorimetric absorbance was measured at 570 nm using an Infinite F200 Pro microplate reader (Tecan), as previously described [7].

### 2.3 Seahorse

hiPSC-cFib were seeded into vitronectin coated Seahorse 24 assay plates at a density of 100 000 cells/well. Oxygen consumption rate (OCR) and extracellular acidification rate (ECAR) were assessed using a standard mitochondrial and glycolysis stress on the Seahorse Bioscience XF-24 analyzer (Agilent technologies). CBD treatment (2 μM) was performed 24 h before measurement. On the day of metabolic flux analysis, cells were washed with Seahorse XF base medium pH 7.4 (Agilent technologies). The culture medium was replaced with 500 μL of Seahorse medium supplemented with 10 mM glucose, 1 mM sodium pyruvate and 2 mM glutamine and incubated 1 h in a CO**_2_**-free incubator at 37 °C. For XF glycolysis stress test, cells were incubated in DMEM with 2 mM glutamine. For the XF Mito stress, inhibitors of the mitochondrial electron transport chain (oligomycin 1 μM, carbonyl cyanide 4-trifluoromethoxy phenylhydrazone (FCCP) 4 μM, rotenone and antimycin 1 μM) were sequentially injected to assess the OCR and the respiratory parameters. For XF glycolysis stress, glucose 10 mM, oligomycin 1 μM and 2-deoxy-d-glucose (2-DG, 100 mM) were injected to evaluate the ECAR and the glycolysis parameters. OCR and ECAR were automatically calculated by the Seahorse XF-24 software, as previously described [7].

### 2.4 Measurement of intracellular reactive oxygen species (ROS) using H_2_DCFDA and mitochondrial oxidative stress using MitoSOX™ Red mitochondrial superoxide indicator

hiPSC-cFib were seeded into vitronectin-coated 96 well plates. Vitronectin was applied at least 1 h prior to cell seeding and cells were plated at a density of 10 000 cells per well. After 24h, serum was withdrawn and cells were incubated with CBD for 24 h. Intracellular ROS levels were evaluated with the fluorescent probe H_2_DCFDA and mitochondrial oxidative stress with MitoSox red (ThemoFisher). A 5 μM working solution of H_2_DCFDA was prepared by diluting a 40 mM stock solution 1:8000 in Hank's Balanced Salt Solution (HBSS). A 5 mM MitoSOX Red, stock solution was prepared by dissolving 50 μg in 15 μL dimethyl sulfoxide (DMSO). The MitoSOX working solution (2.5 μM) was freshly prepared in HBSS briefly vortexed, and protected from light until use. Cells were first washed by removing the culture medium and rinsing with 100 μL of HBSS. Then, 100 μL of the H_2_DCFDA working solution (5 μM in HBSS) for intracellular ROS or 100 μL of the MitoSOX working solution was added to each well. Following a 15 min incubation at room temperature, protected from light, the solution was removed, and cells were washed once more with 100 μL of HBSS. Fresh HBSS (100 μL) was then added to each well, and fluorescence was immediately measured using the Incucyte SX5 Live-Cell Analysis System (Sartorius), utilizing phase contrast, green fluorescence channels (excitation: 453-485 nm; emission: 494–533 nm) for H2DCFDA and orange fluorescence channels (excitation: 546–568 nm; emission: 576–639 nm) for MitoSOX with a 20× objective. Scans were acquired once per condition, and ROS signals were monitored over time. For MitoSOX, images were acquired at multiple time points to assess mitochondrial superoxide-dependent probe oxidation.

### 2.5 Determination of GSH content and the GSSG/GSH ratio

The intracellular levels of reduced glutathione (GSH) and oxidized glutathione (GSSG) were measured using a commercial GSH/GSSG Assay Kit (HY-KO0311, MedChemExpress, USA) according to the manufacturer's instructions. hiPSC-cFib were cultured under basal conditions and treated for 24h in 0.5% SVF with CBD at concentrations of 0.5 μM and 2.0 μM. Following treatment, cells were harvested and lysed in the provided extraction buffer. Lysates were centrifuged to remove debris, and supernatants were collected for analysis. The assay is based on the reaction of GSH with 5,5′-dithiobis-(2-nitrobenzoic acid) (DTNB), forming a yellow-colored product measurable at 412 nm. Total glutathione was measured directly, while GSSG was quantified after masking endogenous GSH using the provided scavenging reagent. Absorbance was measured and standard curves were generated using known concentrations of GSH and GSSG standards. Results were normalized to total protein content, determined using a bicinchoninic acid (BCA) assay. The GSSG/GSH ratio was calculated as an indicator of cellular redox status. All samples were analyzed in duplicate.

### *2.6* *Western blot analysis* and flow cytometry

Whole-cell lysates were prepared using RIPA lysis buffer (Cell Signaling, #9806) supplemented with a protease and phosphatase inhibitor cocktail (Sigma-Aldrich and Thermo Fisher Scientific, #78420). Protein concentrations were determined using the BCA Protein Assay Kit (Thermo Fisher Scientific, #23225). Equal amounts of protein were loaded per lane onto 10%-15% polyacrylamide gels and subjected to electrophoresis. The stacking phase was run at 90 V for 10–15 min, followed by the separation phase at 120 V for 45 min to 1 h. Proteins were then transferred onto 0.45 μm PVDF membranes (Immobilon-P, Merck Millipore, REF: IPVH00010), pre-activated in 99.98% ethanol for 5 min, using an electroblotting apparatus (Bio-Rad PowerPac Basic) for 2 h at 300 mA. After transfer, membranes were rinsed with 1× Tris-buffered saline containing 0.1% Tween-20 (TBST) and blocked for 1 h at room temperature in 1× TBST containing 5% bovine serum albumin (BSA; Sigma-Aldrich). After blocking, membranes were washed again with 1× TBST and incubated overnight at 4 °C with the appropriate primary antibodies (Table 1). The following day, membranes were washed with 1× TBST and incubated with appropriate secondary antibody conjugated to horseradish peroxidase for 1 h 30 min at room temperature. Protein detection was performed using the Clarity Western ECL substrate (Bio-Rad), and signals were visualized using the ChemiDoc MP Imaging System (Bio-Rad). Analysis of CB1R and CB2R expression in hiPSC-cFibs was performed using flow cytometry. Briefly, dead cells were excluded by staining hiPSC-cFibs with Live/Dead Yellow (Invitrogen) for 20 min at 4 °C in the dark. Cells were subsequently fixed and permeabilized for 30 min using the Fixation/Permeabilization Concentrate (Invitrogen). After washing with FACS buffer (PBS supplemented with 4% fetal bovine serum (FBS) and 2 mM EDTA), cells were blocked with 4% mouse serum for 15 min. Samples were then incubated for 30 min at 4 °C with APC-conjugated anti-CB1R and Alexa Fluor 594-conjugated anti-CB2R antibodies (R&D Systems) or with the corresponding isotype controls (mouse IgG2a-APC and mouse IgG2b-Alexa Fluor 594). Data were acquired on an LSRFortessa flow cytometer (BD Biosciences) and analyzed using FlowJo software (Tree Star).

**Table 1:** Antibodies used for western blot, immunofluorescence and flow cytometry.

Target	Provider	Ref
α-SMA	Santa Cruz Biotechnology	sc-32251
Collagen type I	Proteintech	14695-1-AP
Collagen type I	Cell Signaling Technology	#72026
HO -1	Proteintech	10701-1-AP
Hsp90 (4F10)	Santa Cruz Biotechnology	sc-69703
LC3B Antibody	Cell Signaling Technology	#2775
LDHA	Santa Cruz Biotechnology	sc-137243
PDHE1a	Santa Cruz Biotechnology	sc-377092
Phospho PDHE1a	Cell Signaling Technology	# 31866
p62, SQSTM1	Proteintech	18420-1-AP
RhoGDI	Santa Cruz Biotechnology	sc-365190
Human Cannabinoid CB1 APC-conjugated	R&D systems	# FAB3834A
Human Cannabinoid CB2 Alexa Fluor® 594-conjugated	R&D systems	# FAB36551T

### 2.7 RNA extraction and real-time quantitative PCR (RT-qPCR)

RT-qPCR was performed as previously described [7]. Total RNA was extracted using the ReliaPrep™ RNA Cell Miniprep System (Promega) according to the manufacturer's instructions. RNA concentration and purity were measured using a NanoDrop spectrophotometer (ND-2000, Thermo Fisher Scientific). Reverse transcription was carried out using the High-Capacity cDNA Reverse Transcription Kit (Thermo Fisher Scientific, #4368814) with equal amounts of RNA.

RT-qPCR reactions were performed using TB Green® Premix Ex Taq™ II (Takara) on a QuantStudio™ 5 Real-Time PCR System (Thermo Fisher Scientific). Gene expression levels were normalized to the housekeeping genes 36B4 and GAPDH. Relative expression was calculated using the 2^−ΔΔCt method. Primer sequences are listed in Table 2.

**Table 2:** Primer sequences.

Gene	Forward Sequence (5′-3′)	Reverse Sequence (5′-3′)
*36B4*	CAGATCACGTCATCGCACAAC	AAAAGGAGGTCTTCTCGGGC
*GAPDH*	AAGGTCGGAGTCAACGGATTT	ATGAAGGGGTCATTGATGGCA
*Acta2 (asma)*	GGAGCAGCCCAGCCAAGC	AGAGCCCAGAGCCATTGTCAC
*CBR1*	TGGTGTATGATGTCTTTGGGAAG	CGTGTCGCAGGTCCTTACT
*CBR2*	GCTCCTCATCTGTTGGTTCC	TGACCATGGAGTTGATGAGGC
*Col1A1*	GAGGGCCAAGACGAAGACATC	CAGATCACGTCATCGCACAAC
*FAAH*	CTGAAGCTTCCCCAATGGCT	GGTACACCTCGATCTCGTGC
*MAGL*	TCCAGCATGCCAGAGGAAAG	TGGGACACAAAGATGAGGGC

### 2.8 Statistical analysis

Data were obtained from three independent DMD patients and healthy donors. Independent iPSC differentiation batches were performed for each biological sample to generate technical replicates**.** Results are presented as the mean ± standard error of the mean (SEM). Statistical analyses were performed using GraphPad Prism version 10 (GraphPad Software, San Diego, CA, USA). For comparison of two groups, unpaired two-tailed Student's *t*-test was applied. For comparison involving three or more groups, one-way analysis of variance (ANOVA) followed by appropriate multiple-comparisons testing. A *P* value < 0.05 was considered statistically significant.

## 3 Results

### 3.1 CBD differentially regulates the cell cycle and metabolic pathway of hiPSC-cFib from DMD patients and healthy controls

**Fig. 1:**
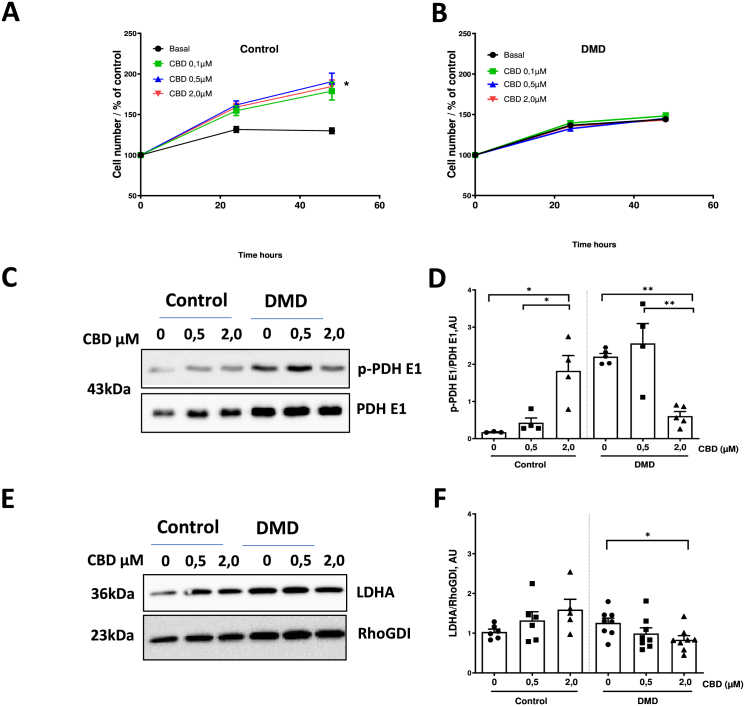
**Effect of CBD on cell number and metabolic protein expression in hiPSC-cFib** **(A–B)** Dose–response effect of cannabidiol (CBD) on hiPSC-cFib from healthy controls (A) and Duchenne muscular dystrophy (DMD) patients (B). Cells were treated with increasing concentrations of CBD (0.1–2 μM) for 24 h to 48h, and cell number was quantified. Data are presented as mean ± SEM from n = 3 healthy donors and n = 3 DMD patients. Statistical differences between basal versus CBD (0.1-2 μM) were determined with a non-parametric *t*-test in control and DMD groups. *P < 0.05 was considered statistically significant **(C–D)** Representative western blots and quantification of phosphorylated pyruvate dehydrogenase E1 (p-PDH E1) and total PDH E1 expression in hiPSC-cFib from control and DMD patients treated with CBD (0.5–2 μM, 24 h). Data are presented as mean ± SEM from n = 3 healthy donors and n = 3 DMD patients, with technical replicates obtained from independent hiPSC-cFib differentiation batches. Statistical differences between treatments in control and DMD groups were determined using a non-parametric one-way ANOVA. *P < 0.05, **P < 0.01. **(E–F)** Representative western blots and quantification of lactate dehydrogenase A (LDHA) expression in hiPSC-cFib from control and DMD patients following CBD treatment (0.5–2 μM, 24 h). Data are presented as mean ± SEM from n = 3 healthy donors and n = 3 DMD patients, with technical replicates derived from independent hiPSC-cFib differentiation batches. Statistical differences between treatments in control and DMD groups were determined using a non-parametric one-way ANOVA. *P < 0.05 versus untreated condition.

Accumulating evidence suggests that CBD regulates cell viability, therefore, we evaluated the dose-dependent effect of CBD on cell number of hiPSC-cFib obtained from DMD patients and healthy controls. At concentrations between 0.1 and 2 μM, CBD significantly increased the number of hiPSC-cFib from healthy controls, while no change was observed in hiPSC-cFib from DMD patients, indicating that the proliferative effect of CBD was restricted to cells derived from healthy controls (Fig. 1A and B). To further determine whether the differential proliferative response to CBD in hiPSC-cFib may be driven by altered activation of metabolic pathways, we examined the expression of the key multienzyme pyruvate dehydrogenase (PDH) complex, which plays a central role in the regulation of glucose homeostasis. The activity of the PDH complex is regulated by reversible phosphorylation of the pyruvate dehydrogenase E1α subunit (PDH E1α), which controls PDH activity and the conversion of pyruvate into acetyl-CoA, thereby fueling the tricarboxylic acid (TCA) cycle and oxidative phosphorylation. We observed a significant increase in PDH E1α phosphorylation in hiPSC-cFib from healthy controls following CBD treatment, whereas hiPSC-cFib from DMD patients exhibited PDH E1α dephosphorylation (Fig. 1C and D). In parallel, expression of lactate dehydrogenase A (LDHA), which catalyzes the conversion of pyruvate to lactate, was slightly increased in control hiPSC-cFib but decreased in DMD hiPSC-cFib upon CBD treatment, suggesting that CBD promotes glycolytic metabolism selectively in cells derived from healthy individuals (Fig. 1E and F).

### 3.2 hiPSC-cFib from DMD patients exhibited an impaired glycolytic response to CBD stimulation

**Fig. 2:**
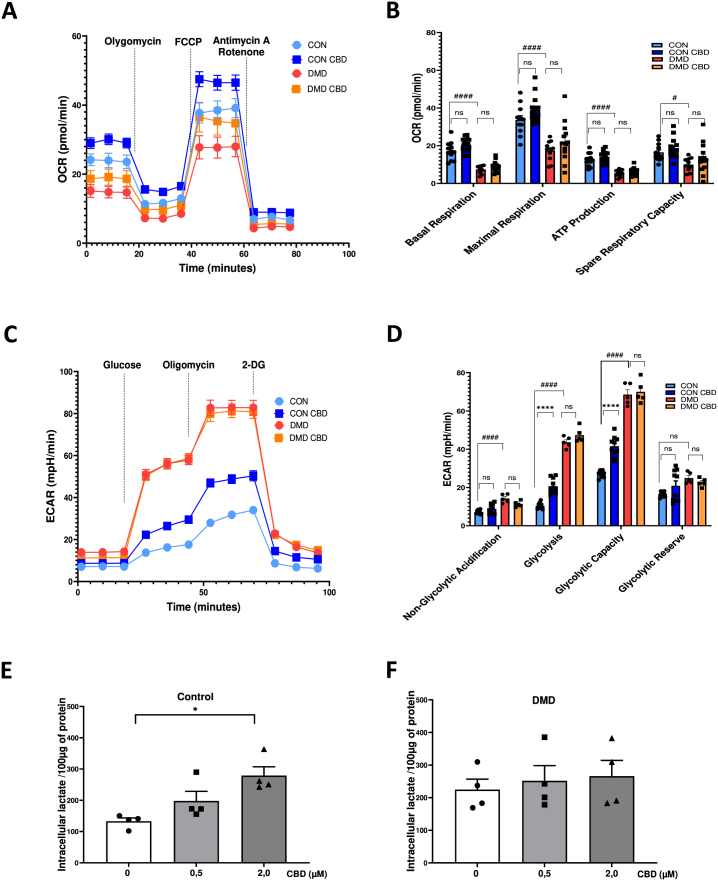
**Effects of CBD on mitochondrial respiration and glycolytic metabolism in hiPSC-cFib.** **(A–B)** Mitochondrial respiration in hiPSC-cFib from healthy controls and DMD patients treated with CBD (2 μM, 24 h). (A) Representative oxygen consumption rate (OCR) traces. (B) Quantification of basal respiration, maximal respiration, ATP-linked respiration, and spare respiratory capacity. OCR was assessed under basal conditions and following sequential injections of oligomycin (1 μM), FCCP (4 μM), and rotenone/antimycin A (1 μM each). Data are presented as mean ± SEM from n = 3 healthy donors and n = 3 DMD patients, with technical replicates obtained from independent hiPSC-cFib differentiation batches. Statistical differences between treatments in control and DMD groups and differences between control and DMD groups were determined using a non-parametric one-way ANOVA. A value of *P < 0.05 was considered statistically significant**.** (****P < 0.0001, CBD vs basal; ####P < 0.0001, control vs DMD; ns, not significant). **(C–D)** Glycolytic function in hiPSC-cFib from healthy controls and DMD patients treated with CBD (2 μM, 24 h). (C) Representative extracellular acidification rate (ECAR) traces. (D) Quantification of non-glycolitic acidification, glycolysis, glycolytic capacity, and glycolytic reserve. ECAR was measured under basal conditions and after sequential addition of glucose (10 mM), oligomycin (1 μM), and 2-deoxy-d-glucose (2-DG, 100 mM). Data are presented as mean ± SEM from n = 3 healthy donors and n = 3 DMD patients, with technical replicates of biological samples obtained from independent hiPSC-cFib differentiation batches. Statistical differences between treatments in control and DMD groups and differences between control and DMD groups were determined using one-way ANOVA. A value of *P < 0.05 was considered statistically significant. (****P < 0.0001, basal vs CBD; ####P < 0.0001, control vs DMD; ns, not significant). **(E–F)** Intracellular lactate levels in hiPSC-cFib from healthy controls (E) and DMD patients (F) following CBD treatment (0.5–2 μM, 24 h). Data are presented as mean ± SEM from three healthy donors and three DMD patients, with technical replicate derived from independent hiPSC-cFib differentiation batches. Statistical differences between treatment conditions in the control and DMD groups were determined using a non-parametric one-way ANOVA. *P < 0.05 versus untreated condition.

To more precisely assess the impact of CBD on cellular metabolism, mitochondrial respiration and glycolytic function were quantified using a Seahorse analyzer. Measurement of oxygen consumption rates (OCR) indicated that basal and maximal respiration, ATP production and spare respiratory capacity were not significantly changed following CBD treatment, although a tendency toward increased respiration was observed in both hiPSC-cFib from healthy controls and DMD patients (Fig. 2A and B). In contrast, the rate of acidification in hiPSC-cFib healthy controls evaluated by extracellular flux glycolysis stress test was significantly increased demonstrating substantial improvement in glycolysis and glycolytic capacity induced by CBD. Unexpectedly, CBD failed to stimulate glycolysis in hiPSC-cFib from DMD patients, as both glycolysis and glycolytic capacity remained unchanged, indicating that CBD could not enhance glycolytic flux in these cells (Fig. 2C and D). Given that hiPSC-cFib DMD cells exhibit pre-existing metabolic reprogramming toward glycolysis, we stimulated the cells with TGF-β, which is known to induce glycolysis, to verify whether glycolysis could still be induced in hiPSC-cFib DMD cells. Unlike CBD, TGF-β could increase glycolysis and glycolytic capacity in both control and DMD hiPSC-cFibs (Supplementary Fig. 1). The induction of glycolysis by CBD in controls hiPSC-cFib was further corroborated by an increase in intracellular lactate levels (Fig. 2E and F). Collectively, these data demonstrate a differential effect of CBD on glycolytic pathways in hiPSC-cFib from healthy controls versus DMD patients, which may account for the genotype-dependent differences observed in CBD-induced cell proliferation.

### 3.3 CBD modestly reversed the myofibroblast phenotype of DMD hiPSC-cFib independently of metabolism

**Fig. 3:**
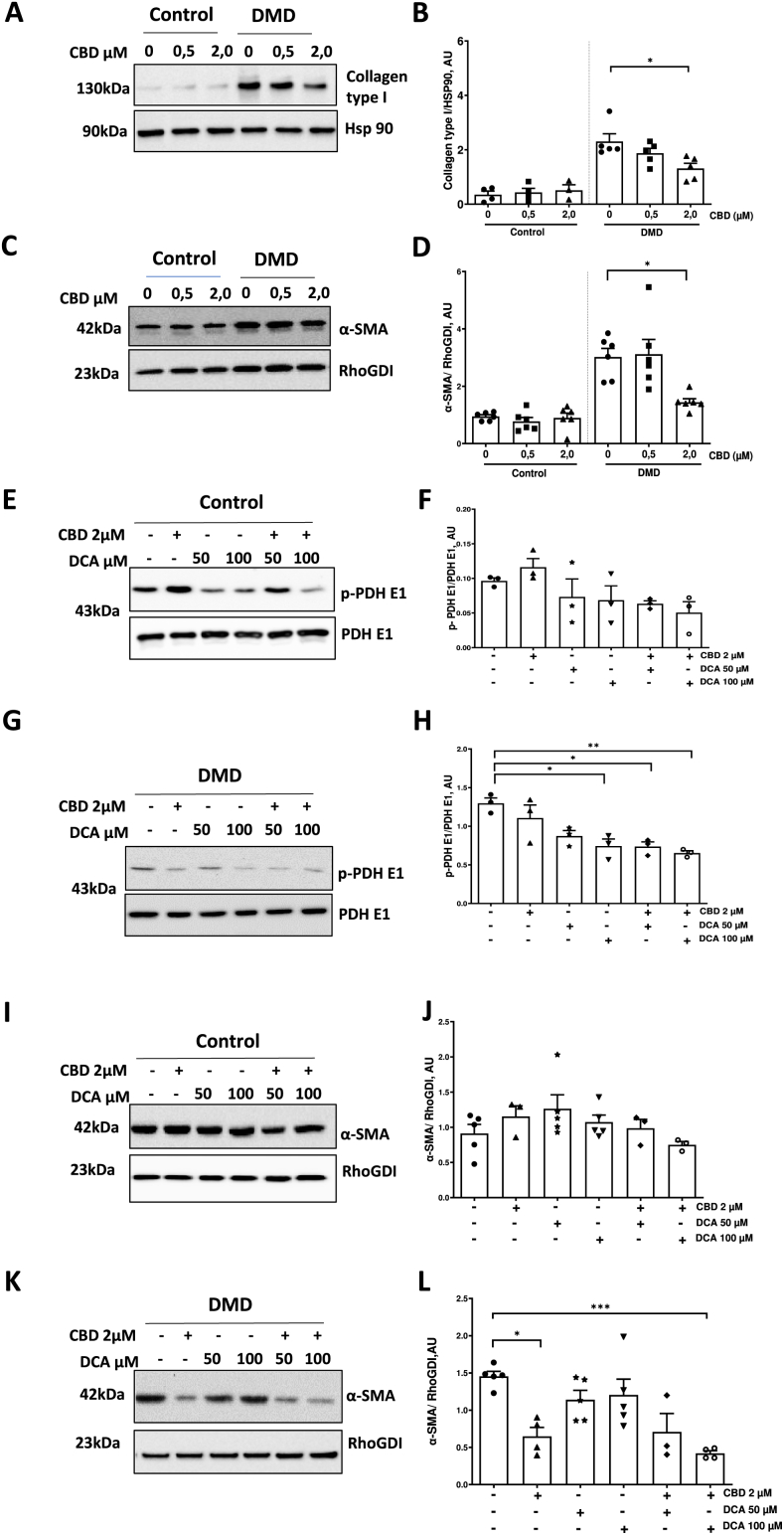
**Effects of CBD and dichloroacetate (DCA) on fibrotic and metabolic protein markers in hiPSC-cFib.** **(A–B)** Representative western blots and quantification of collagen type I and α-smooth muscle actin (α-SMA) **(C–D)** in hiPSC-cFib derived from healthy controls and DMD patients treated with CBD (0.5–2 μM, 24 h). Data are presented as mean ± SEM from n = 3 healthy donors and n = 3 DMD patients, with technical replicate derived from independent hiPSC-cFib differentiation batches performed on each biological sample. Statistical differences between treatments in control and DMD groups were determined using a non-parametric one-way ANOVA. P < 0.05 was considered statistically significant**.** **(E–H)** Representative western blots and quantification of p-PDH E1 and total PDH E1 in hiPSC-cFib derived from healthy controls (E–F) and DMD patients (G–H) treated with CBD (2 μM) and DCA (50–100 μM, 24 h). Data are presented as mean ± SEM from n = 3 healthy donors and n = 3 DMD patients. Statistical differences between treatments in control and DMD groups were determined using a non-parametric one-way ANOVA. *P < 0.05, **P < 0.01. **(I–L)** Representative western blots and quantification of α-SMA in hiPSC-cFib derived from healthy controls (I–J) and DMD patients (K–L) treated with CBD (2 μM) and DCA (50–100 μM, 24 h). Data are presented as mean ± SEM from n = 3 healthy donors and n = 3 DMD patients, with technical replicate obtained from independent hiPSC-cFib differentiation batches. Statistical differences between treatments in control and DMD groups were determined using a non-parametric one-way ANOVA. *P < 0.05, ***P < 0.001.

Since a primary function of fibroblasts is the regulation of the extracellular matrix, we investigated whether CBD treatment alters the phenotype of hiPSC-cFib by assessing the expression of protein markers associated with myofibroblast differentiation. Under basal conditions, CBD treatment of hiPSC-cFib from DMD patients resulted in a modest but significant downregulation of collagen type I and of the contractile protein α-smooth muscle actin (α-SMA), as determined by western blot analysis (Fig. 3A–D). To evaluate whether modulation of PDH by CBD contributes to fibroblast phenotype changes, we incubated the cells with sodium dichloroacetate (DCA), a small molecule inhibitor of pyruvate dehydrogenase kinases that impairs PDH phosphorylation. As expected, DCA downregulated the p-PDH E1/PDH E1 ratio in hiPSC-cFib from both DMD patients and healthy controls (Fig. 3E–H), but did not modify the level of α-SMA expression (Fig. 3I–L). Moreover, while CBD did not further inhibit PDH phosphorylation in DMD hiPSC-cFib, it still downregulated α-SMA, indicating that CBD attenuates the activated phenotype of DMD hiPSC-cFib independently of pyruvate metabolism regulation.

### 3.4 CBD reverses the TGF-β–induced activation of DMD hiPSC-cFib by a mechanism largely independent of cannabinoid receptors

**Fig. 4:**
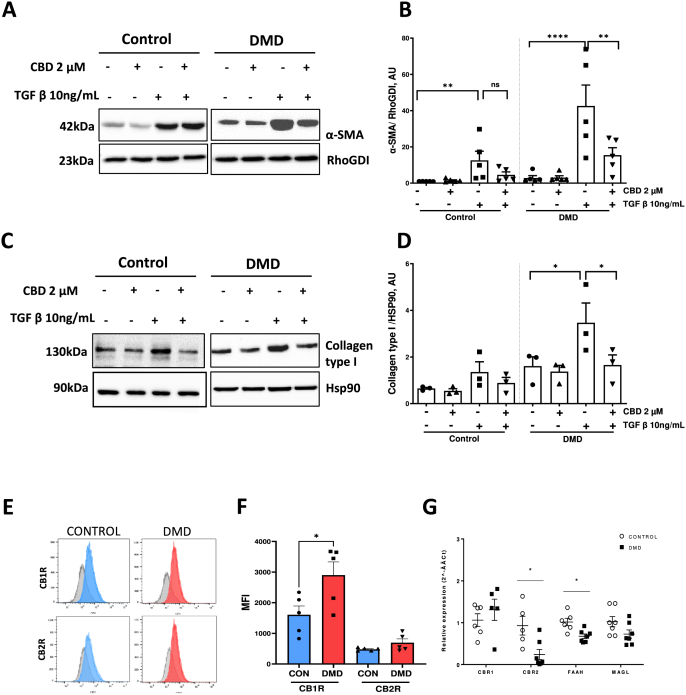
**Effect of CBD on profibrotic activation and endocannabinoid signaling in hiPSC-cFib.** **(A–B)** Representative western blot and quantification of α-SMA in hiPSC-cFib from healthy controls and DMD patients treated with CBD (2 μM) in the presence of TGF-β stimulation (10 ng/mL, 24 h). Data are presented as mean ± SEM from n = 3 healthy donors and n = 3 DMD patients, with technical replicates obtained from independent hiPSC-cFib differentiation batches. Statistical differences between treatments in control and DMD groups were determined using a non-parametric one-way ANOVA. **P < 0.01, ****P < 0.0001 were considered statistically significant. **(C–D)** Representative western blot and quantification of collagen type I protein in hiPSC-cFib from healthy controls and DMD patients under the same treatment conditions. Data are presented as mean ± SEM from three healthy donors and three DMD patients, with technical replicates derived from independent hiPSC-cFib differentiation batches. Differences between treatment conditions in the control and DMD groups were evaluated using a non-parametric one-way ANOVA *P < 0.05 vs untreated condition. **(E–F)** Representative flow cytometry plots and quantification of mean fluorescence intensity (MFI) showing basal expression of endocannabinoid receptors in hiPSC-cFib from control and DMD patients. Data are presented as mean ± SEM from n = 3 healthy donors and n = 3 DMD patients, with a technical replicate of biological sample obtained from independent hiPSC-cFib differentiation batches. Statistical differences between control and DMD groups were determined using a non-parametric *t*-test. *P < 0.05 versus healthy donors group. **(G)** mRNA levels of key endocannabinoid receptors (*CB1R*, *CB2R*) and metabolic enzymes (*FAAH*, *MAGL*) in hiPSC-cFib from control and DMD patients. Data are presented as mean ± SEM from n = 3 healthy donors and n = 3 DMD patients, with technical replicates generated from independent hiPSC-cFib differentiation batches. Statistical differences between control and DMD groups were determined using a non-parametric *t*-test. *P < 0.05 versus healthy donors group.

Given our previous findings that hiPSC-cFib from DMD patients exhibit high sensitivity to the profibrotic factor TGF-β, we further examined the effects of CBD in the presence of TGF-β stimulation. Treatment with TGF-β induced the expression of the profibrotic markers α-SMA and collagen type I in both DMD and control hiPSC-cFib compared with basal conditions. Notably, CBD attenuated TGF-β–induced expression of α-SMA and collagen type I proteins in both DMD and controls hiPSC-cFib, as assessed by western blotting (Fig. 4A–D). To further investigate the involvement of endocannabinoid receptors in CBD's protective effect, we first assessed the expression of key components of the endocannabinoid system, including receptors and metabolic enzymes. The endocannabinoid receptor CB1R, previously reported to be upregulated in the muscles of DMD patients [14], was likewise over represented in hiPSC-cFib from Duchenne patients, whereas CB2R receptor levels were unchanged (Fig. 4E and F). However, gene expression analysis revealed a downregulation of CB2R as well as fatty acid amide hydrolase (FAAH), the enzyme responsible for the hydrolysis of the endocannabinoid anandamide (AEA), in DMD hiPSC-cFib compared with controls (Fig. 4 G). These results show that the dysregulation of the endocannabinoid system reported in DMD patients is also present in hiPSC-cFib.

**Fig. 5:**
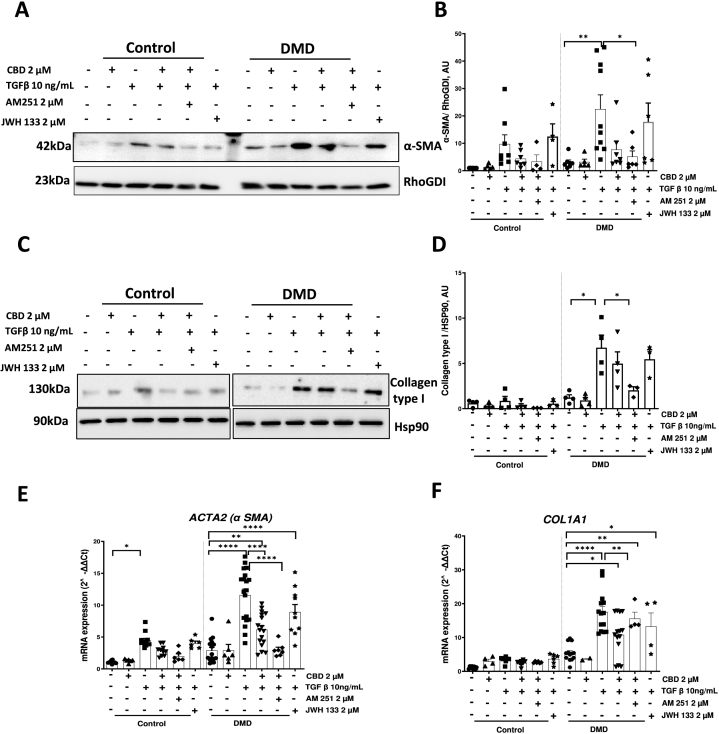
**CB1R and CB2R involvement in CBD-mediated modulation of TGF-β–induced profibrotic markers in hiPSC-cFib.** **(A–B)** Representative western blot and quantification of α-SMA in hiPSC-cFib from healthy controls and DMD patients treated with CBD (2 μM) in the presence of TGF-β (10 ng/mL, 24 h) and CB1/CB2 modulators (2 μM AM251 or 2 μM JWH133, 24 h). Data are presented as mean ± SEM from n = 3 healthy donors and n = 3 DMD patients with technical replicates of biological sample obtained from independent hiPSC-cFib differentiation batches. Statistical differences between treatments in control and DMD groups were determined using a non-parametric one-way ANOVA. *P < 0.05, **P < 0.01. **(C–D)** Representative western blot and quantification of collagen type I protein expression under the same conditions. Data are presented as mean ± SEM from n = 3 healthy donors and n = 3 DMD patients with technical replicates generated from independent hiPSC-cFib differentiation batches. Statistical differences between treatments in control and DMD groups were determined using a non-parametric one-way ANOVA. *P < 0.05. **(E–F)** mRNA levels of *ACTA2* (*α-SMA)* (E) and *COL1A1* (F) in hiPSC-cFib from control and DMD patients treated under the same conditions. Data are presented as mean ± SEM from n = 3 healthy donors and n = 3 DMD patients, with technical replicates of the biological samples obtained from independent hiPSC-cFib differentiation batches. Statistical differences between treatments in control and DMD groups were determined using a non-parametric one-way ANOVA. *P < 0.05, **P < 0.01, ****P < 0.0001.

The contribution of CB1R to the inhibitory effect of CBD was evaluated using the CB1R antagonist AM251. The presence of AM251 did not abolish the inhibitory effect of CBD on TGF-β–induced α-SMA and collagen type I expression, but instead AM251 induced a slight reduction in α-SMA and collagen type I expression. To assess whether CB2R activation could recapitulate CBD's effects, hiPSC-cFib were treated with the CB2R agonist JWH-133**.** Treatment with JWH-133 did not significantly alter TGF-β–induced α-SMA and collagen type I expression (Fig. 5A–D). Consistent with these observations, gene expression analyses confirmed that modulation of endocannabinoid receptors minimally modified CBD-mediated downregulation of TGF-β–induced profibrotic genes *ACTA2 (α-SMA)* and *COL1A1* (Fig. 5E and F), indicating that the effects of CBD are partially independent of endocannabinoid receptor signaling.

### 3.5 CBD-induced autophagy fails to reverse the myofibroblast phenotype in DMD hiPSC-cFib

**Fig. 6:**
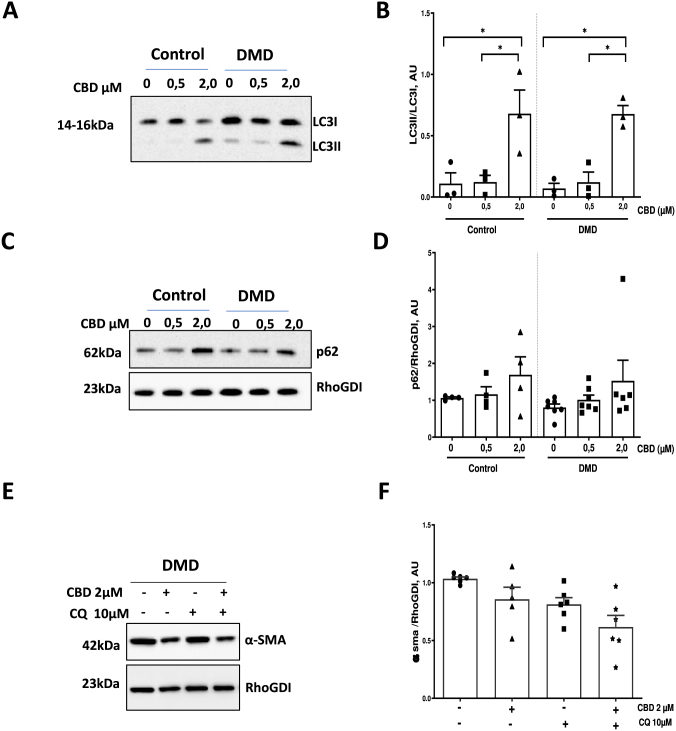
**CBD reverses the myofibroblast phenotype in hiPSC-cFib from DMD patients independently of metabolic and autophagy regulation.** **(A–B)** Representative western blots and quantification of the LC3B-II/LC3B-I ratio and p62 **(C–D)** in hiPSC-cFib derived from healthy controls and DMD patients treated with CBD (0.5–2 μM, 24 h). Data are presented as mean ± SEM from n = 3 healthy donors and n = 3 DMD patients, with technical replicates of biological samples obtained from independent hiPSC-cFib differentiation batches. Statistical differences between treatments in control and DMD groups were determined using a non-parametric one-way ANOVA. *P < 0.05. **(E–F)** Representative western blots and quantification of α-SMA protein expression in hiPSC-cFib from DMD patients treated with CBD (2 μM), chloroquine (CQ, 10 μM). Data are presented as mean ± SEM from three healthy donors and three DMD patients, with technical replicates derived from independent hiPSC-cFib differentiation batches of biological samples. Statistical differences between conditions in DMD group were determined using a non-parametric one-way ANOVA.

As several studies suggest that cellular autophagy, a nutrient recycling pathway, is a player of fibroblast-to-myofibroblast differentiation [22], we first evaluated autophagy pathways by examining the conjugation of microtubule-associated protein light chain 3 (LC3) to phosphatidylethanolamine and the expression of the cargo protein p62. At basal level, hiPSC**-**cFib from DMD patients and healthy donors showed comparable level of LC3BII/LC3I and p62 expression. Treatment with 2 μM CBD increased the LC3BII/I ratio in hiPSC-cFib from both DMD patients and healthy donors, while p62 levels rose slightly (Fig. 6A–D). These findings suggest that CBD induced weak autophagy in hiPSC-cFib, regardless of genotype. To assess the potential involvement of autophagy in the CBD-mediated attenuation of fibroblast activation, hiPSC-cFib were exposed to the autophagy inhibitor chloroquine (CQ) that impairs the final step of autophagosome-lysosome fusion. Blocking autophagy with CQ led to accumulation of p62 (Supplementary Fig. 2) and slightly reduced α-SMA expression (Fig. 6E and F). CBD-induced inhibition of α-SMA expression was maintained in the presence of CQ indicating that CBD action was not hindered by the inhibition of the autophagic flux suggestive of an alternative mechanism of action.

### 3.6 CBD triggers antioxidant pathways in hiPSC-cFib from DMD patients and healthy controls

**Fig. 7:**
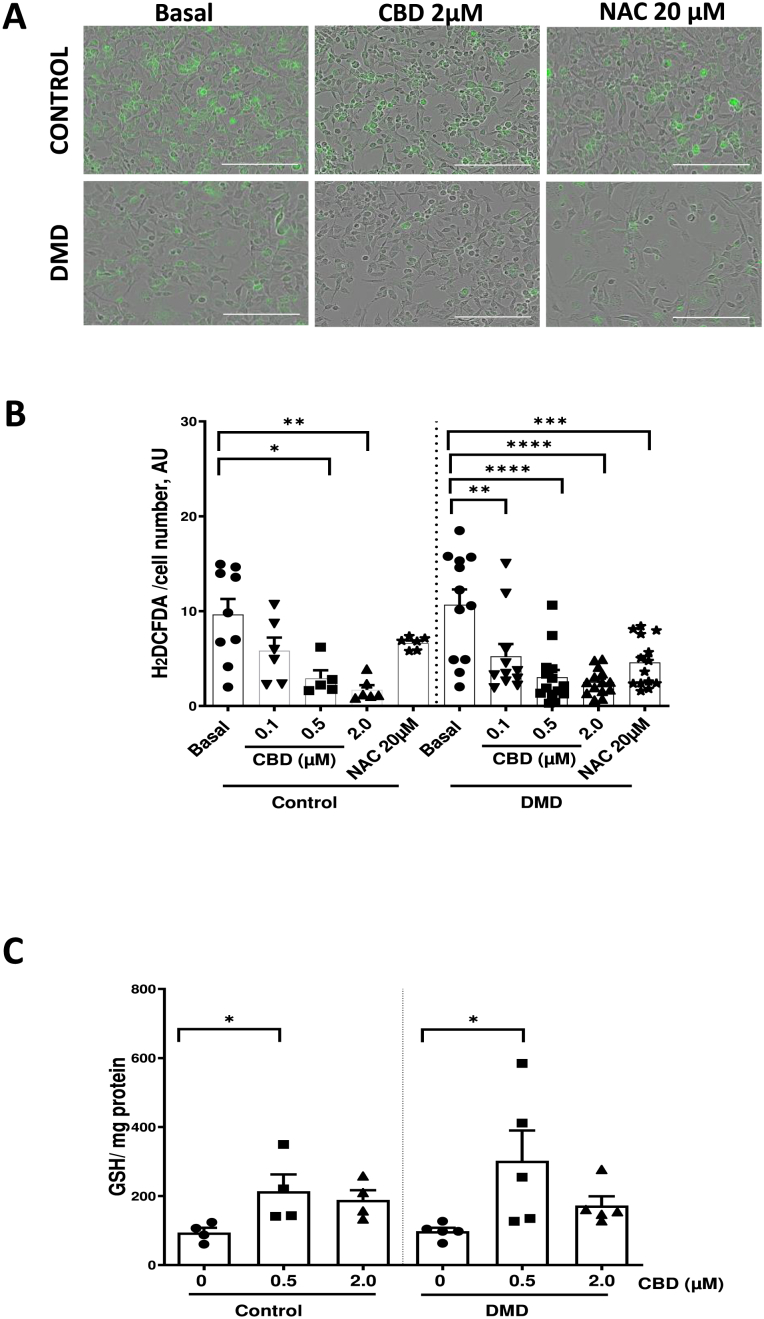
**CBD activates antioxidant pathways in hiPSC-cFib from DMD patients and healthy controls.** **(A)** Representative H_2_DCFDA fluorescence images of hiPSC-cFib from healthy controls and DMD patients following treatment with CBD (0.1–2 μM) or N-acetylcysteine (NAC, 20 μM) for 24 h. Scale bar: 200 μm **(B)** Quantification of intracellular reactive oxygen species (ROS) levels assessed by H2DCFDA fluorescence in hiPSC-cFib treated with different doses of CBD or NAC (20 μM), in both DMD and healthy control derived cells. Data are presented as mean ± SEM from three healthy donors and three DMD patients, with technical replicates per biological sample obtained from independent hiPSC-cFib differentiation batches. Statistical differences between conditions in control and DMD groups were determined using a non-parametric one-way ANOVA. *P < 0.05,**P < 0.01,****P < 0.0001. **(C)** Quantification of glutathione (GSH) levels in hiPSC-cFib from healthy controls and DMD patients following treatment with CBD (0.5–2 μM) for 24h. Data are presented as mean ± SEM from n = 3 healthy donors and n = 3 DMD patients with technical replicates obtained from independent hiPSC-cFib differentiation batches of biological samples. Statistical differences between treatments in control and DMD groups up were determined using a non-parametric one-way ANOVA. *P < 0.05**.**

Oxidative stress-induced ROS play a critical role in Duchenne muscular dystrophy and CBD has been widely described to have anti-oxidative proprieties. Therefore, we first evaluated the level of ROS using the H_2_DCFDA fluorescence probe for cytosolic ROS measurement. Interestingly, fluorescence generated by H_2_DCFDA oxidation was not significantly different in hiPSC-cFib from DMD patients and healthy controls (Fig. 7A and B). CBD elicited a dose-dependent reduction in H_2_DCFDA fluorescence that exceeded the effect observed with 20 μM N-acetylcysteine (NAC) in both DMD and control hiPSC-cFib, demonstrating a potent antioxidant effect of CBD (Fig. 7A and B). CBD significantly elevated intracellular GSH levels, both in control and DMD hiPSC-cFib and tend to decrease GSSG/GSH ratio, consistent with an enhancement of antioxidant capacity (Fig. 7C, Supplementary Fig. 3). In contrast, at basal level, CBD had no effect on MitoSOX staining both in DMD and control hiPSC-cFib, indicating that mitochondrial superoxide was not affected by CBD treatment (Supplementary Fig. 4).

**Fig. 8:**
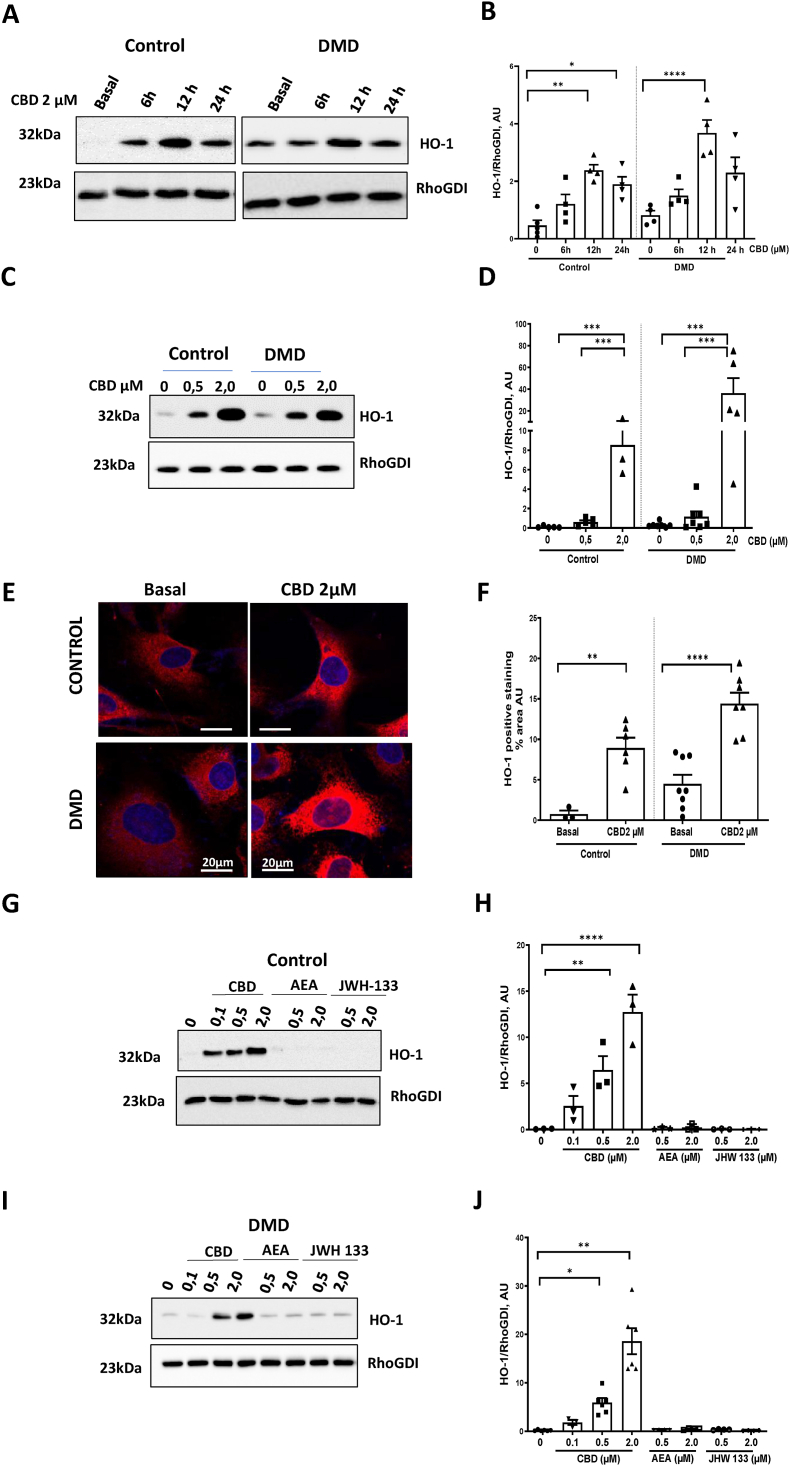
**CBD up-regulates heme oxygenase-1 (HO-1) protein expression in hiPSC-cFib from DMD patients and healthy controls.** **(A–B)** Representative western blots and quantification of HO-1 protein expression in hiPSC-cFib from healthy controls and DMD patients treated with CBD (2 μM) over a time course (6, 12, and 24 h). Data are presented as mean ± SEM from n = 3 healthy donors and n = 3 DMD patients, with technical replicates obtained from independent hiPSC-cFib differentiation batches. Statistical differences between treatments in control and DMD groups were determined using a non-parametric one-way ANOVA. *P < 0.05, **P < 0.01, ****P < 0.0001. **(C–D)** Representative western blots and quantification of HO-1 protein expression in hiPSC-cFib from healthy controls and DMD patients treated with increasing concentrations of CBD (0.5–2 μM) for 24 h (dose-response). Data are presented as mean ± SEM from n = 3 healthy donors and n = 3 DMD patients, with technical replicates of biological sample obtained from independent hiPSC-cFib differentiation batches. Statistical differences between treatments in control and DMD groups were determined using a non-parametric one-way ANOVA. *P < 0.05,***P < 0.001. **(E–F**) Representative 63x confocal images of hiPSC-cFib stained for HO-1 (red) and nuclei (DAPI, blue). Scale bar: 20 μm (E) and quantification of HO-1 fluorescence intensity (F). Data are presented as mean ± SEM from n = 3 healthy donors and n = 3 DMD patients, with technical replicates obtained from independent hiPSC-cFib differentiation batches. Statistical differences between treatments in control and DMD groups were determined using a parametric *t*-test. **P < 0.01, ****P < 0.0001 for CBD versus basal condition. **(G–H)** Representative western blots and quantification of HO-1 in hiPSC-cFib from healthy controls treated with CBD (0.1–2 μM, 24 h), AEA, or JWH-133 (0.5–2 μM, 24 h). Data are presented as mean ± SEM from n = 3 healthy donors and n = 3 DMD patients, with a technical replicate obtained from independent hiPSC-cFib differentiation batches. Statistical differences between treatments in control and DMD groups were determined using a non-parametric one-way ANOVA. **P < 0.01, ****P < 0.0001 versus basal condition. **(I–J)** Representative western blots and quantification of HO-1 in hiPSC-cFib from DMD patients under the same treatment conditions. Data are presented as mean ± SEM from n = 3 healthy donors and n = 3 DMD patients, with technical replicates per biological sample obtained from independent hiPSC-cFib differentiation batches. Statistical differences between treatments in control and DMD groups were determined using a non-parametric one-way ANOVA. *P < 0.05,**P < 0.01 versus basal condition.

**Fig. 9:**
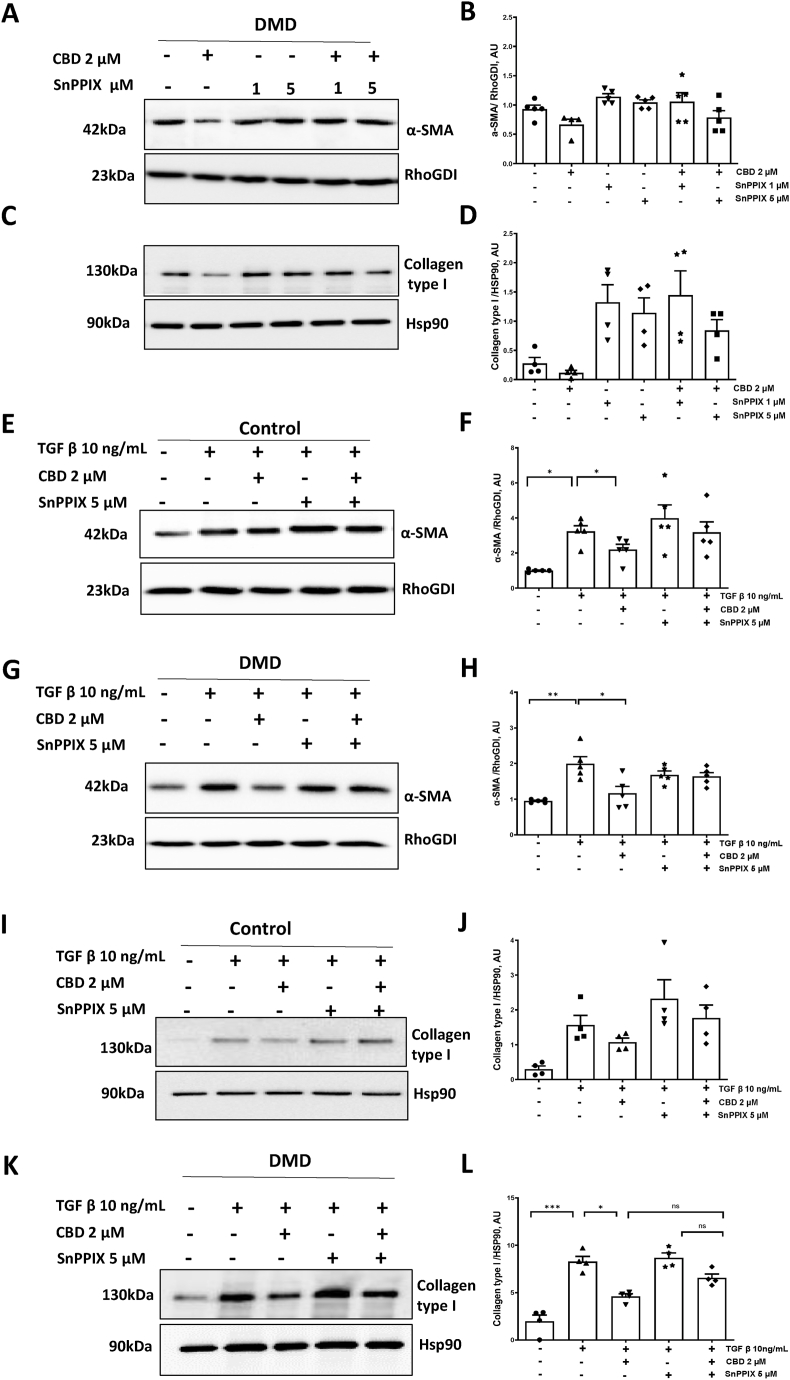
**Anti-fibrotic effects of CBD require HO-1 activity.** **(A–B)** Representative western blots and quantification of α-SMA, and **(C–D)** collagen type I in hiPSC-cFib from DMD patients treated with CBD (2 μM) in the presence or absence of the HO-1 inhibitor SnPPIX (1 or 5 μM) for 24 h. Data are presented as mean ± SEM from three healthy donors and three DMD patients with technical replicates obtained from independent hiPSC-cFib differentiation batches. Statistical differences between treatments in DMD group were evaluated using a non-parametric one-way ANOVA**.** **(E–H)** Representative western blots and quantification of α-SMA in hiPSC-cFib from healthy controls (E–F) and DMD patients (G–H) treated with CBD (2 μM, 24 h) in the presence of TGF-β stimulation (10 ng/mL, 24 h) and SnPPIX (5 μM, 24 h). Data are presented as mean ± SEM from n = 3 healthy donors and n = 3 DMD patients, with technical replicates of biological sample obtained from independent hiPSC-cFib differentiation batches. Statistical differences between treatments in control and DMD groups were determined using a non-parametric one-way ANOVA. *P < 0.05,**P < 0.01. **(I–L)** Representative western blots and quantification of collagen type I in hiPSC-cFib from healthy controls (I–J) and DMD patients (K–L) under the same treatment conditions. Data are presented as mean ± SEM from n = 3 healthy donors and n = 3 DMD patients, with technical replicates of biological sample obtained from independent hiPSC-cFib differentiation batches. Statistical differences between treatments in control and DMD groups were determined using a non-parametric one-way ANOVA. *P < 0.05,***P < 0.001.

To elucidate the mechanisms underlying CBD-mediated ROS suppression, we examined HO-1 expression, an antioxidant enzyme that is dysregulated in myogenic cells of patients with DMD [23]. HO-1 expression was low under basal conditions in both DMD and control hiPSC-cFib and was strongly induced by CBD in a time- and dose-dependent manner (Fig. 8A–D). Immunofluorescence analysis confirmed a significant increase in cytoplasmic HO-1 protein following CBD treatment (Fig. 8E and F). In contrast, no induction of HO-1 protein was detected in the presence of the synthetic CB2R agonist JWH-133 or the CB1R agonist AEA, although CB1R and CB2R agonists elicited a modest but significant increase in the proliferation of DMD and control hiPSC-cFibs respectively (Fig. 8G–J, Supplementary Fig. 5). Furthermore, the CB1R and CB2R antagonists, AM251 and AM630 respectively, failed to inhibit CBD-induced HO-1 upregulation, suggesting that cannabidiol-mediated induction of HO-1 occurs largely independently of cannabinoid receptor signaling (Supplementary Fig. 6). Previous studies have demonstrated that ROS contribute to collagen synthesis in cardiac fibroblasts [24,25]. To investigate whether HO-1 mediates the antifibrotic effect of CBD, hiPSC-cFib were treated with the HO-1 inhibitor tin protoporphyrin IX (SnPPIX). Inhibition of HO-1 slightly increased basal expression of α-SMA and collagen type I in DMD hiPSC-cFib and blunted the inhibitory effect of CBD under basal conditions (Fig. 9A–D). Moreover, SnPPIX did not modify TGFβ -induced expression of α-SMA and collagen type I in hiPSC-cFib from DMD patients or healthy controls, but abolished CBD-mediated inhibition of TGFβ-induced fibroblast activation, indicating that HO-1 is required for the antifibrotic effect of CBD (Fig. 9E–L). These findings demonstrate that CBD's mechanism of action is largely dependent on HO-1 induction. Collectively, these data demonstrate that HO-1 induction contributed to the antifibrotic effect of CBD and could be considered as promising target for attenuation of cardiac fibrosis and remodeling during Duchenne cardiomyopathies.

## 4 Discussion

The loss of dystrophin causes muscle damage and fibro fatty replacement in both skeletal muscle and heart. Modulation to the endocannabinoid system has shown benefits in locomotor performance of dystrophic mdx making the endocannabinoid system a therapeutical target for treatment of Duchenne patients [17,24]. This study evaluated the effect of cannabidiol on hiPSC-cFib. Interestingly, we observed that CBD substantially increased glycolysis in hiPSC-cFib from healthy controls, through regulation of PDH E1 phosphorylation and conversion of pyruvate to lactate. Glycolysis is well known to promote cell proliferation by providing anabolic substrates, and CBD-induced stimulation of glycolysis may promote cell growth in control hiPSC-cFib cells. The absence of an effect of CBD on glycolysis and cell proliferation in hiPSC-cFibs derived from patients with DMD may be attributable to pre-existing metabolic reprogramming toward glycolysis, as previously described [7]. Further research is needed to clarify the dysregulated mechanisms in DMD hiPSC-cFibs that impede CBD-induced glycolysis. However, it is likely that compensatory signaling pathways activated by dystrophin loss overlap with CBD-induced glycolytic signaling, which could explain the loss of CBD effects in DMD hiPSC-cFibs. It is interesting to note that TGF-β-induced glycolysis stimulation was maintained in both control hiPSC-cFibs and DMD hiPSC-cFibs, further reinforcing the fact that CBD-induced modulation of glycolysis was not involved in its attenuating effect on TGF-β-induced hiPSC-cFib activation. Interest in CBD initially stemmed from its pharmacological modulation of the endocannabinoid system and its ability to reduce inflammation and fibrosis in different experimental disease models [26,27]. Our findings **show** that the antifibrotic action of CBD occurs largely independently of the canonical endocannabinoid receptors CB1R and CB2R but dependent on the antioxidant enzyme HO-1. In the heart, HO-1 has been revealed as cardioprotective during ischemia reperfusion conditions by preventing oxidative stress and apoptosis [28,29]. HO-1 degrades heme and thus protects cells from heme-induced oxidative damage. The enzyme localizes in the cytoplasm, anchored to the endoplasmic reticulum but is also described in other cell compartments, including the nucleus where it protects cells from replicative stress [[30], [31], [32]]. For control hiPSC-cFibs and DMD hiPSC-cFibs, CBD treatment resulted in an increase in HO-1 in the cell cytoplasm, suggesting a low impact on proliferative effect. In the mdx model of DMD, inhibition of HO-1 led to increased inflammation and exacerbated skeletal muscle damage [33]. A central role of HO-1 in the attenuation of fibrosis has been revealed in different organs such as heart, lung and liver [34]. More recently, deletion of HO-1 in zebrafish was sufficient to significantly increased cardiac fibrosis and collagen deposition without cardiac injury suggesting a homeostatic role in cardiac tissue [35]. In addition, an essential role of the enzyme in fibroblasts homeostasis was highlighted by a myofibroblast-restricted overexpression of HO-1 that was shown to ameliorate liver fibrosis without altering liver injury in mice [36]. Our data identified HO-1 as a key mediator contributing to the antifibrotic effects of CBD, the involvement of HO-1 in the inhibitory effect of CBD was primarily demonstrated through pharmacological inhibition and potential off-target effects of HO-1 inhibitor cannot be ruled out. Moreover, the contribution of endocannabinoid receptor signaling in HO-1 induction cannot be excluded, however it appeared limited as, neither the endogenous endocannabinoid ligand AEA nor the synthetic CB2R agonist JWH-133 stimulated HO-1 expression. Furthermore, the CB1R and CB2R antagonists failed to prevent CBD-induced HO-1 expression in hiPSC-cFibs derived from Duchenne patients or healthy donors under the concentrations and time frames examined. Nevertheless, the involvement of cannabinoid receptors in the antifibrotic actions of CBD cannot be entirely excluded, as cannabinoid receptor signaling appears to be highly dependent on organ context and cell type. Indeed, CB2R have been shown to mediate the protective effects of cannabidiol against stress-induced liver inflammation and fibrosis, whereas CB1R activation exacerbates fibrogenesis within the same tissue [37,38]. The peculiarity of the phytocannabinoid CBD to stimulate HO-1 independently of endocannabinoid receptors was also reported in adipose tissue-derived mesenchymal stem cells [3]. In these cells, CBD enhanced cell survival through the induction of autophagy whereas HO-1 contributed to apoptosis under conditions where autophagy was disrupted [39].

Our study revealed that hiPSC-cFib from DMD patients have lost metabolic response to CBD compared with healthy controls. In contrast, cannabidiol exerted a protective effect against the over-activation of Duchenne hiPSC-derived fibroblasts associated with HO-1 induction, highlighting its therapeutic potential to mitigate cardiac fibrosis associated with Duchenne muscular dystrophy.

## Ethics declaration

### Informed consent and patient details

Written informed consent to take part in the study and to publish the article has been obtained from all participants or their legal representatives. The privacy rights of participants have been observed.

### Human and biological material

This study included organ or tissue donors. This study includes human biological material and consent was obtained by donors, or their next of kin or legal representatives, for use in this study and for publication of the article. The samples used in this research were not sourced from executed prisoners or prisoners of conscience.

### Studies in Human

This study was performed in compliance with relevant laws, regulatory frameworks and guidelines where the research took place. This study was approved by the Centro Cardiologico Monzino IRCCS. (Approval No. Local Ethical Committee (06/06/2012)).

## Declaration of generative AI use

During the preparation of this work, the authors used OpenAI's ChatGPT to assist with minor language improvements, including grammar and readability.

## Funding

This work was supported by grant from the 10.13039/501100020407European Research Area Network on Cardiovascular Diseases (JTC2018-046 DENIM). L.S. was supported by grants from ANR- PAUSE and the FRM-PAUSE (Fondation pour la Recherche Médicale).

## CRediT authorship contribution statement

**L. Savchenko:** Conceptualization, Data curation, Formal analysis, Investigation, Methodology, Writing – original draft, Writing – review & editing. **S. Soussi:** Data curation, Investigation. **D. Rovina:** Methodology, Resources. **A. Swiader:** Investigation, Methodology. **S. Mallia:** Resources. **G. Pompilio:** Funding acquisition. **O. Kunduzova:** Funding acquisition. **A. Parini:** Funding acquisition. **A. Gowran:** Conceptualization, Funding acquisition, Project administration, Resources. **N. Pizzinat:** Conceptualization, Data curation, Formal analysis, Funding acquisition, Validation, Writing – original draft, Writing – review & editing.

## **Declaration of competing interest**

The authors declare that they have no known competing financial interests or personal relationships that could have appeared to influence the work reported in this paper.

## Data Availability

Data will be made available on request.
