# Supplementary material for: Cannabidiol-induced Heme oxygenase-1 contributes to modulate the phenotype of hiPSC-derived cardiac fibroblasts from patients with Duchenne muscular dystrophy
Source: Redox Biol. 2026 Aug 5;96:104333. doi: 10.1016/j.redox.2026.104333 (PMC13487608; doi:10.1016/j.redox.2026.104333)

Supplementary Figure 1

A

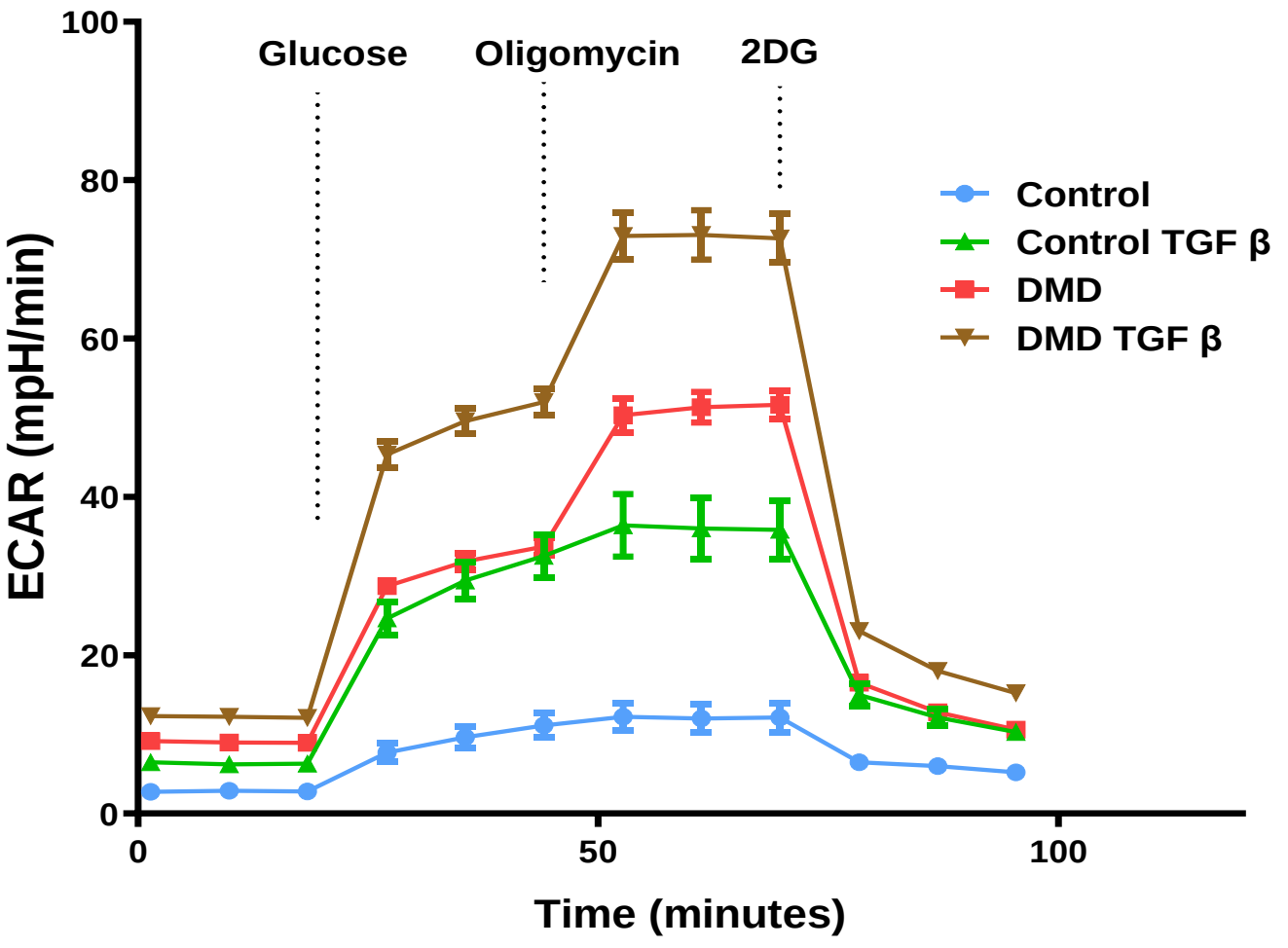

B

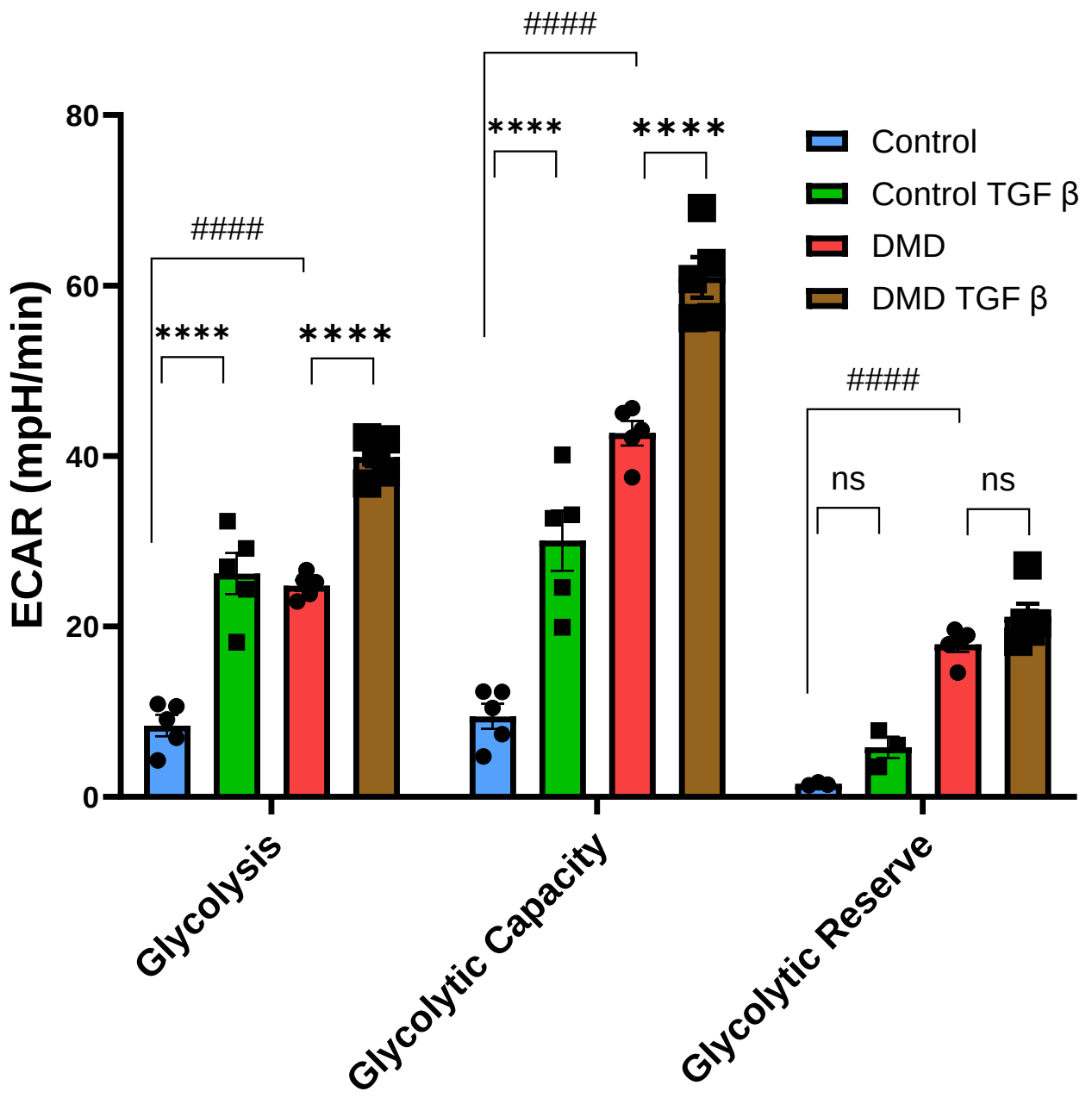

Supplementary Figure 2

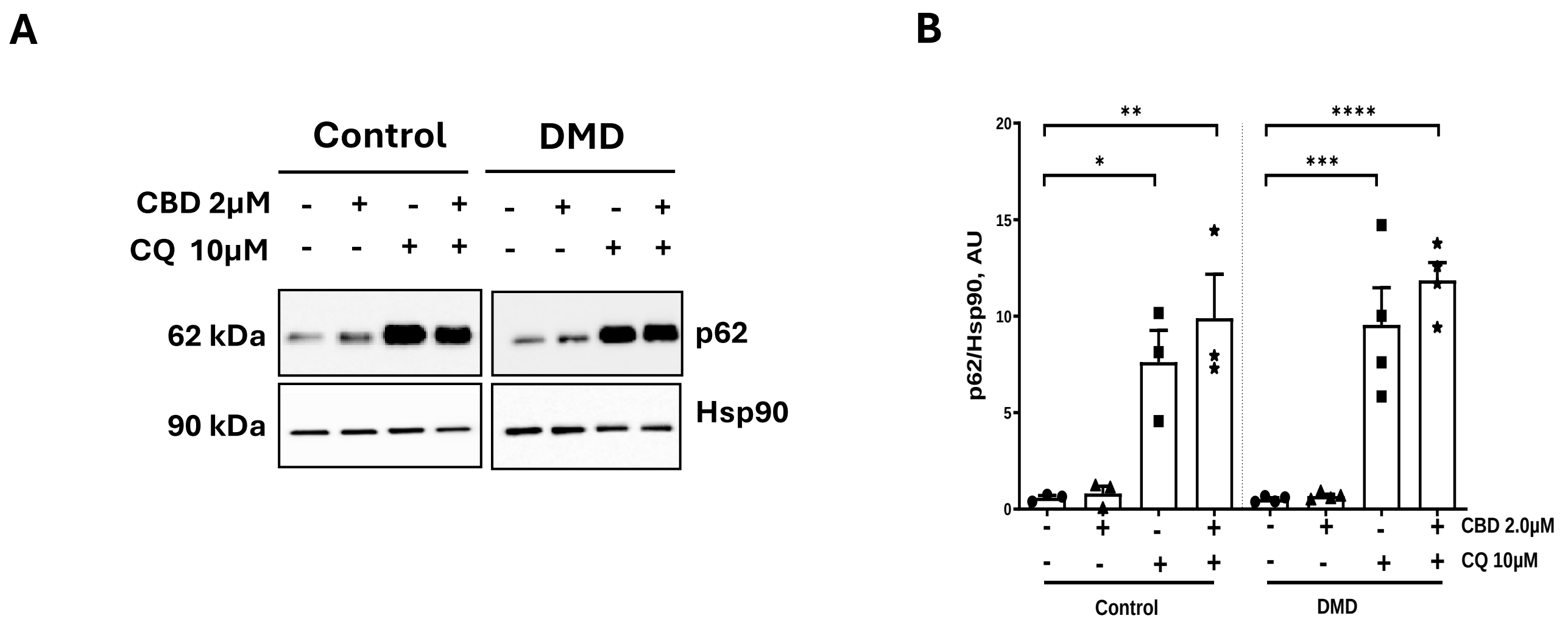

Supplementary Figure 3

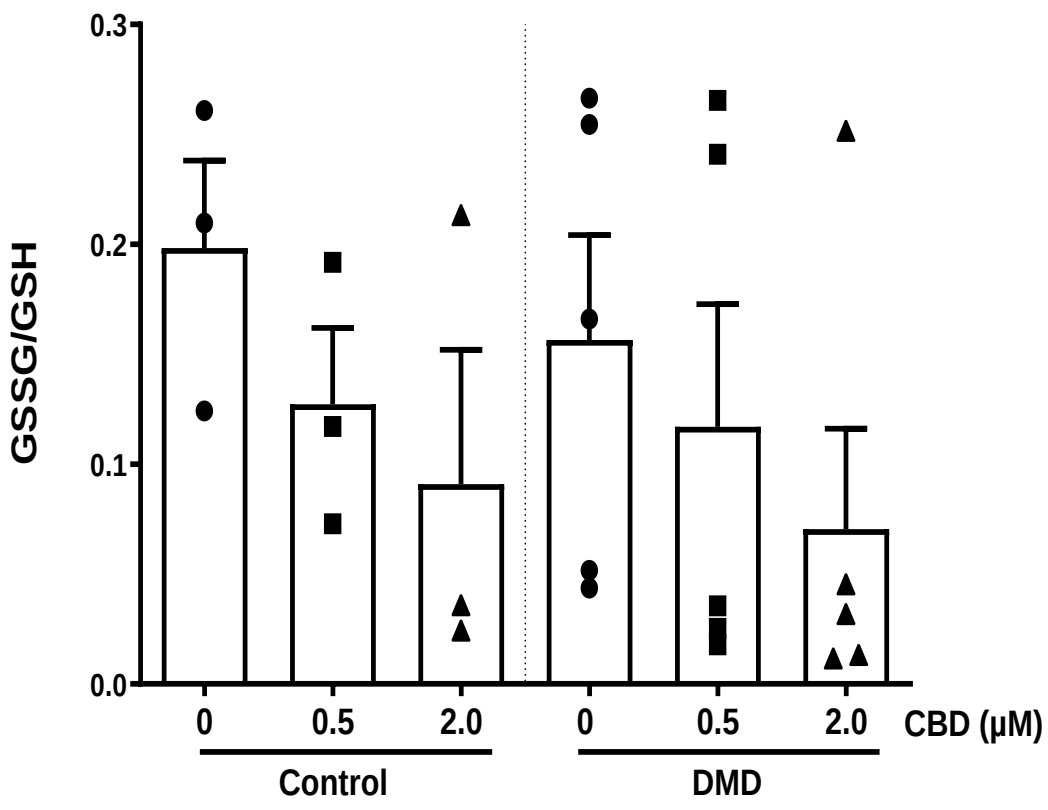

Supplementary Figure 4

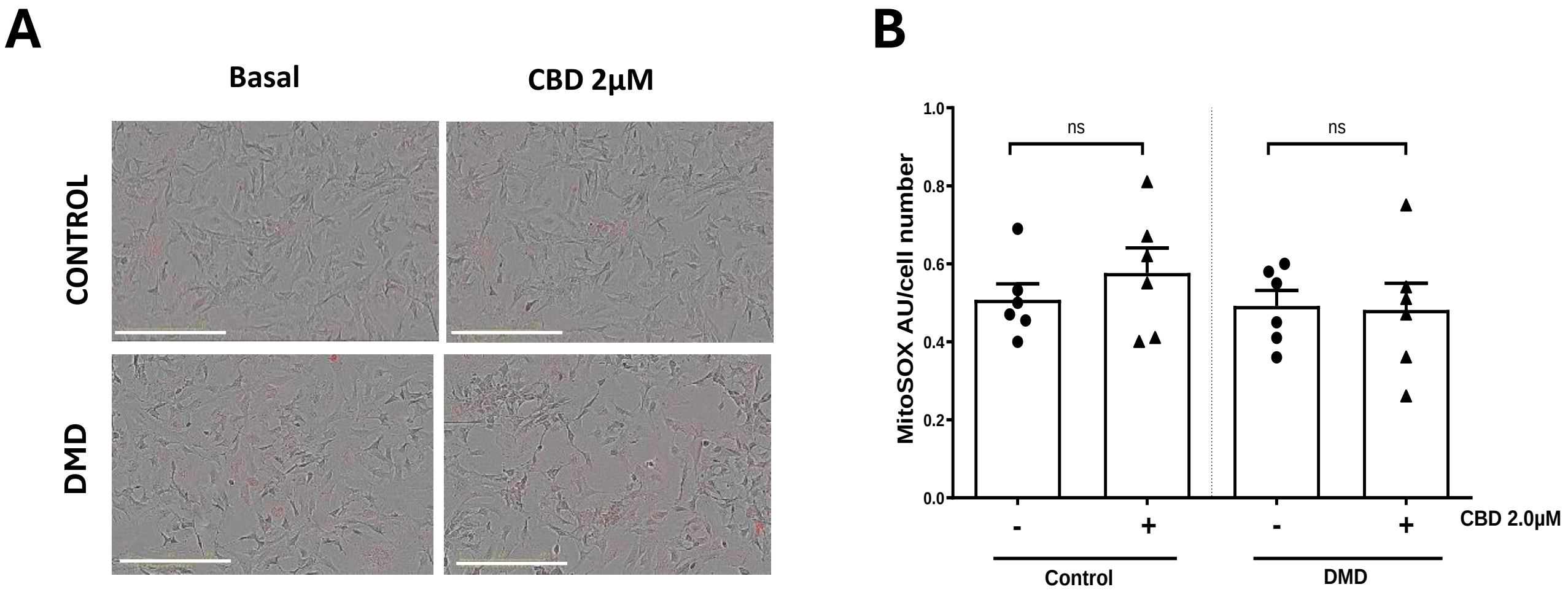

Supplementary Figure 5

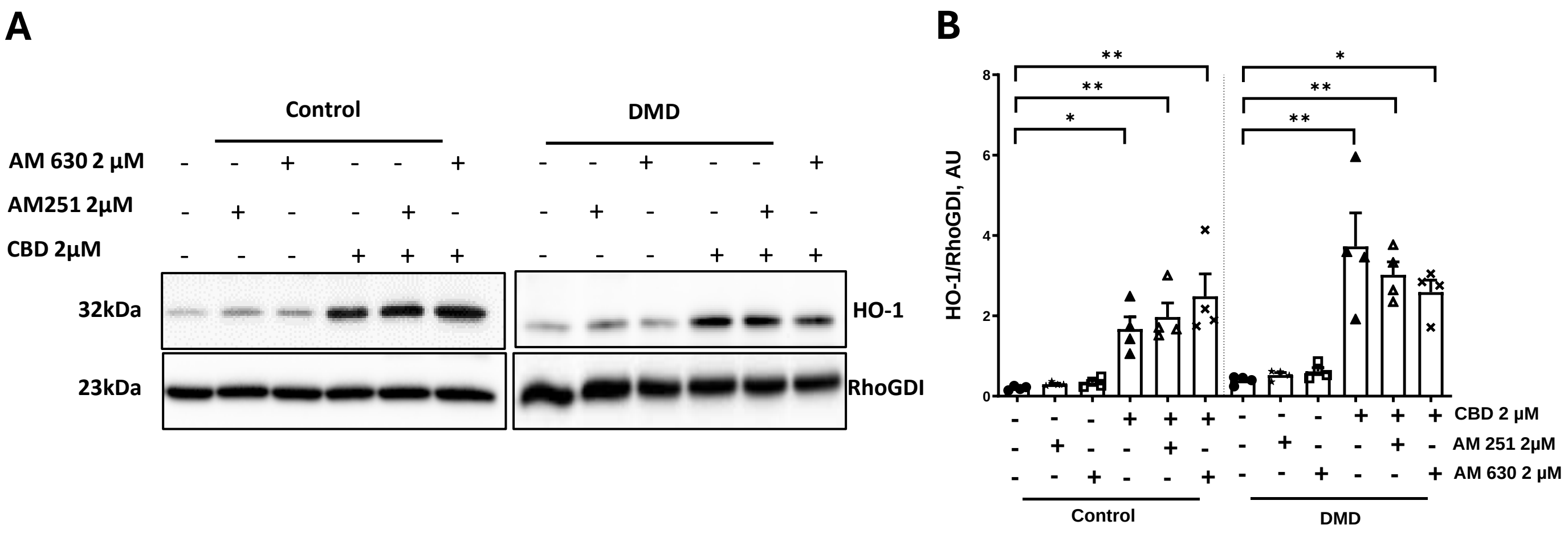

Supplementary Figure 6

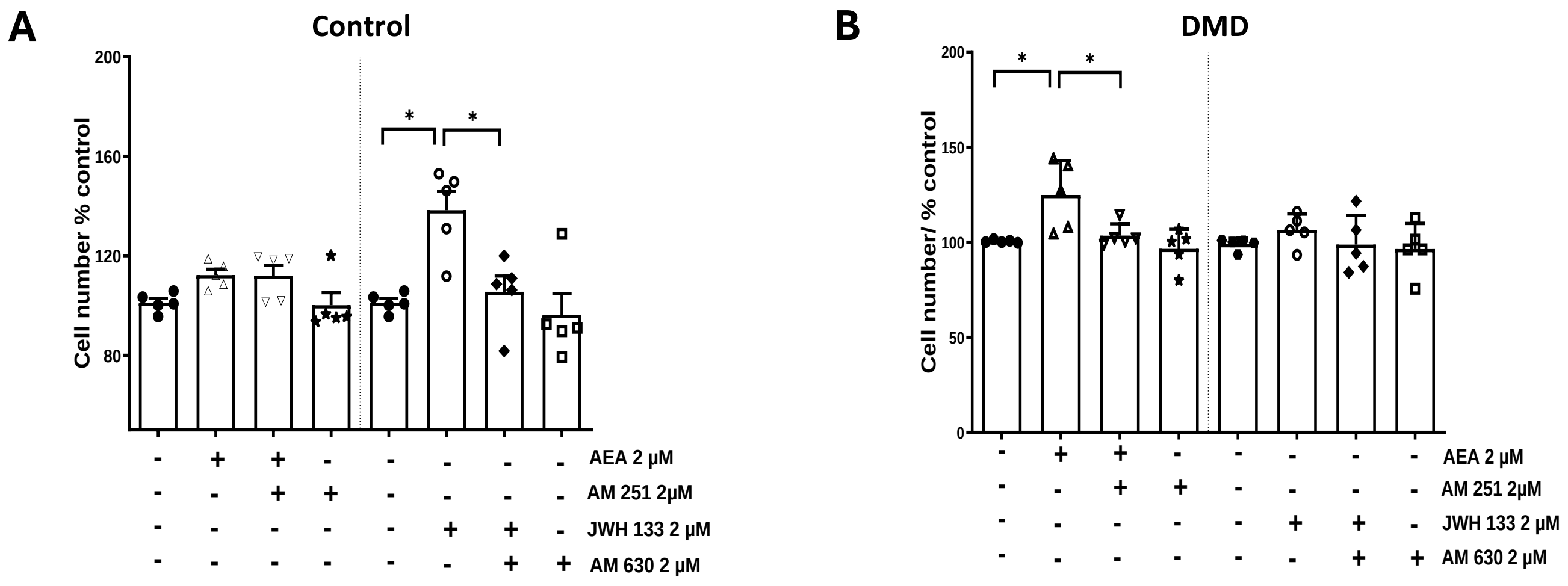

Supplement: Supplementary file 1 [file mmc2.pdf]
