# Supplementary material for: Cannabidiol-induced Heme oxygenase-1 contributes to modulate the phenotype of hiPSC-derived cardiac fibroblasts from patients with Duchenne muscular dystrophy
Source: Redox Biol. 2026 Aug 5;96:104333. doi: 10.1016/j.redox.2026.104333 (PMC13487608; doi:10.1016/j.redox.2026.104333)
Supplement: Supplementary file 2 [file mmc1.docx]

**Supplementary Figure 1: Effects of TGF β on glycolytic metabolism in hiPSC-cFib.** **(A-B)** Glycolytic function in hiPSC-cFib from healthy controls and DMD patients with stimulation TGF β (10 ng/mL, 24 h). **(A)** Representative extracellular acidification rate (ECAR) traces. **(B)** Quantification of glycolysis, glycolytic capacity, and glycolytic reserve. ECAR was measured under basal conditions and after sequential addition of glucose (10 mM), oligomycin (1 µM), and 2-deoxy-D-glucose (2-DG, 100 mM).

Data are presented as mean ± SEM from three healthy donors and three DMD patients, with technical replicates derived from independent hiPSC-cFib differentiation batches. Statistical differences between treatments in control and DMD groups were determined using one-way ANOVA. A value of *P < 0.05 was considered statistically significant. (******P < 0.0001, basal vs** TGF β**; ####P < 0.0001, control vs DMD; ns, not significant).**

**Supplementary Figure 2:** **Effect of CBD and chloroquine on p62 expression in hiPSC-cFib.** **(A)** Representative western blots and **(B)** quantification of p62 in hiPSC-cFib derived from healthy controls and DMD patients treated with CBD (2 µM) or chloroquine (CQ, 10 µM) for 24h. Data are presented as mean ± SEM from three healthy donors and three DMD patients, with technical replicates derived from independent hiPSC-cFib differentiation batches of biological samples. Differences between treatment conditions in the control and DMD groups were evaluated using a non-parametric one-way ANOVA. * P < 0.05, **P < 0.01, ***P < 0.001, ****P < 0.0001.

**Supplementary Figure 3: GSSG/GSH ratios in hiPSC-cFib from healthy controls and DMD patients following 24 h treatment with CBD (0.5–2 µM).** Data are presented as mean ± SEM from three healthy donors and three DMD patients, with technical replicates derived from independent hiPSC-cFib differentiation batches. Statistical differences between treatments in control and DMD groups were determined using a non-parametric one-way ANOVA.

**Supplementary Figure 4: CBD effects on mitochondrial oxidative stress in hiPSC-cFib. (A)** Representative MitoSOX™ Red fluorescence images of hiPSC-cFib derived from healthy controls and DMD patients following treatment with CBD (2 µM) for 24 h. Scale bar: 200 µm. **(B)** Quantification of MitoSOX fluorescence in hiPSC-cFib from control and DMD groups treated with CBD. Data are presented as mean ± SEM from three healthy donors and three DMD patients with one technical replicate per biological sample obtained from independent hiPSC-cFib differentiation batches. Statistical differences between treatments in control and DMD groups were determined using a non-parametric one-way ANOVA. ns: non-significant.

**Supplemental Figure 5: Effects of CBD on HO-1 expression in hiPSC-cFib. (A)** Representative western blots and **(B)** quantification of HO-1 protein expression in hiPSC-cFib from healthy controls and DMD patients treated with CBD (2 µM), AM251 (2 µM) or AM630 (2 µM) for 24 h. Data are presented as mean ± SEM from three healthy donors and three DMD patients, with technical replicates derived from independent hiPSC-cFib differentiation batches. Statistical differences between treatments in control and DMD groups were determined using a non-parametric one-way ANOVA. *P < 0.05, **P < 0.001 treatment vs basal.

**Supplemental Figure 6: Quantification of cell number in hiPSC-cFib.** **(A)** Cell number quantification in hiPSC-cFib derived from healthy controls and **(B)** DMD patients following treatment with CBD in the presence or absence of AEA (2 µM), AM251(2 µM), JWH133 (2 µM), or AM630 (2 µM) for 24 h. Data are presented as mean ± SEM from three healthy donors and three DMD patients, with technical replicates derived from independent hiPSC-cFib differentiation batches. Statistical differences between treatments in control and DMD groups were determined using a non-parametric one-way ANOVA. *P < 0.05 **was considered statistically significant.**
